# HLA-BAT1 alters migration, invasion and pro-inflammatory cytokines in prostate cancer

**DOI:** 10.3389/fonc.2022.969396

**Published:** 2022-11-23

**Authors:** Aileen M. García-Vargas, Yarelis M. Roque-Reyes, Desiree M. Arroyo-Villegas, Daniel Santiago-Negron, María M. Sánchez-Vázquez, Alejandro Rivera-Torres, Andrea C. Reyes-Meléndez, Valerie Cardona-Berdecía, Miosotis García-Maldonado, Olga M. Víquez, Magaly Martínez-Ferrer

**Affiliations:** ^1^ Department of Pharmacology and Toxicology, School of Medicine, University of Puerto Rico, Medical Sciences Campus, San Juan, PR, United States; ^2^ Division of Cancer Biology, University of Puerto Rico Comprehensive Cancer Center, San Juan, PR, United States; ^3^ Department of Biology, University of Puerto Rico, Rio Piedras Campus, San Juan, PR, United States; ^4^ Department of Chemistry, University of Puerto Rico, Rio Piedras Campus, San Juan, PR, United States; ^5^ Department of Pharmaceutical Sciences, School of Pharmacy, University of Puerto Rico, Medical Sciences Campus, San Juan, PR, United States; ^6^ Research Biobank, University of Puerto Rico Comprehensive Cancer Center, San Juan, PR, United States; ^7^ Division of Nephrology and Hypertension, Department of Medicine, Vanderbilt University, Nashville, TN, United States

**Keywords:** prostate cancer, animal models, migration, invasion, inflammation, cytokines

## Abstract

Prostate cancer (PCa) accounts for more than 1 in 5 diagnoses and is the second cause of cancer-related deaths in men. Although PCa may be successfully treated, patients may undergo cancer recurrence and there is a need for new biomarkers to improve the prediction of prostate cancer recurrence and improve treatment. Our laboratory demonstrated that HLA-B-associated transcript 1 (BAT1) was differentially expressed in patients with high Gleason scores when compared to low Gleason scores. BAT1 is an anti-inflammatory gene but its role in PCa has not been identified. The objective of this study is to understand the role of BAT1 in prostate cancer. *In vitro* studies showed that BAT1 down-regulation increased cell migration and invasion. In contrast, BAT1 overexpression decreased cell migration and invasion. RT-PCR analysis showed differential expression of pro-inflammatory cytokines (TNF-α and IL-6) and cell adhesion and migration genes (MMP10, MMP13, and TIMPs) in BAT1 overexpressed cells when compared to BAT1 siRNA cells. Our *in vivo* studies demonstrated up-regulation of TNF-α, IL-6, and MMP10 in tumors developed from transfected BAT1 shRNA cells when compared to tumors developed from BAT1 cDNA cells. These findings indicate that BAT1 down-regulation modulates TNF-α and IL-6 expression which may lead to the secretion of MMP-10 and inhibition of TIMP2.

## 1 Introduction

Prostate cancer (PCa) accounts for more than 1 in 5 diagnoses of cancer in the United States and is the second leading cause of cancer-related deaths in men in the country (1). PCa may be successfully treated with radical prostatectomy, radiation therapy, and other treatments (2). However, patients may undergo cancer recurrence. There is a need for the development of new biomarkers to improve the prediction of cancer recurrence and treatment. Previous findings in our laboratory demonstrated that Human Leukocyte Antigen (HLA)-B Associated Transcript 1 (BAT1) or DDX39B was differentially expressed in patients with high Gleason scores when compared to low Gleason scores. The characterization of biomarkers in PCa may lead to a better prognosis and outcome for PCa patients.

BAT1 is a member of the DEAD-box family of RNA-dependent ATPases. BAT1 is located on the short arm of chromosome 6 in the region of the major histocompatibility complex (MHC) III and is about ~40kb telomeric of tumor necrosis factor-alpha (TNF-α) (3). Its role in splicing is to transfer pre-mRNA from the nucleus to the cytoplasm (4). Studies have shown that unidentified MHC class III genes play a role in inflammatory and immunopathological responses in diseases such as ulcerative colitis, diabetes, rheumatoid arthritis, and multiple sclerosis (5–12). Specifically, HLAs that are located on chromosome 6 and are part of the major histocompatibility complex are highly polymorphic in humans leading to increase immune response through inflammatory cytokine expression (12–14). BAT1 was identified as an anti-inflammatory gene through the modulation of pro-inflammatory cytokines tumor necrosis factor-alpha (TNF-α), interleukin-1 (IL-1), and interleukin-6 (IL-6) in diabetes, Chagas cardiomyopathy, and Plasmodium vivax malaria. Patients that had Chagas cardiomyopathy or Plasmodium vivax malaria and presented polymorphisms in the -22C/G and -348C/T promoter region of BAT1, lead to the increased expression on pro-inflammatory cytokines, specifically, TNF-α and IL-6 when compared to patients that did not have polymorphisms in the promoter region of BAT1 (15–20). The presence of inflammatory components plays a pivotal role in the cancer tumor microenvironment and favors metastasis/cell invasion thus, promoting oncogenesis. The identification of BAT1 as an anti-inflammatory gene in other types of diseases suggests that BAT1 is playing a role in immune response (21). The objective of this study was to identify the biological role of BAT1 in prostate cancer.

In this study, we evaluated the role of BAT1 in migration, invasion, and inflammation using *in vitro* and *in vivo* models of PCa. We demonstrated that BAT1 down-regulation leads to an increase in PCa cell migration and invasion *in vitro*. In contrast, BAT1 overexpression decreased PCa cell migration and invasion *in vitro*. We studied alternative pathways associated with migration and invasion. We focused on matrix metalloproteinases (MMPs) and tissue inhibitors of matrix metalloproteinases (TIMPs). MMP dysregulation in cancer leads to the degradation of the extracellular matrix (ECM) during tissue remodeling and inflammation, which leads to metastasis (22–24). MMPs are proteolytic enzymes that remodel the microenvironment and are present during an event of wound healing and inflammation (25–27). MMPs are highly expressed in diverse types of cancer including breast, prostate, bronchial, and squamous cell carcinoma (28–32). Studies have shown that MMPs are expressed in different prostatic cell types, suggesting that the proteolytic axis during prostate cancer invasion and metastasis is coordinated by stromal and epithelial components (29–31). TIMPs are natural inhibitors of MMPs and their loss of inhibition leads to tumor progression and inflammation in PCa (32, 33). MMPs have been found to be involved in epithelial-mesenchymal transition (EMT). EMT is the transition from epithelial cells to mesenchymal cells and it is characterized by the changes in cell morphology that decrease adhesiveness caused by rearrangements of the cytoskeletal system of tumor cells and this allows them to invade and metastasize surrounding tissues. In addition, it has been shown that EMT is related to the invasion and metastasis ability of tumors in the liver, breast, prostate, and colorectal cancer (32). In our study, BAT1 overexpression decreased TNF-α, IL-6, and MMP mRNA expression when compared to BAT1 down-regulation in PCa cells *in vitro*. Concurrently, BAT1 overexpression increased TIMP RNA expression when compared to BAT1 down-regulation in PCa cells *in vitro*. *In vivo* studies demonstrated that BAT1 down-regulation increased TNF-α, IL-6, and MMP-10 expression. These findings suggest that BAT1 down-regulation causes inflammation and promotes cell migration and invasion. BAT1 overexpression may decrease PCa progression through the modulation of inflammation and migration. BAT1 may serve as a potential biomarker for PCa recurrence.

## 2 Materials and methods

### 2.1 I*n vitro* experiments

#### 2.1.1 Cell culture

Human prostate cancer cell lines, 22RV1 and PC3 obtained from American Type Culture Collection (ATCC, VA, USA) were cultured in RPMI-1640 medium (Hyclone, Waltham, MA, USA) containing 5% penicillin-streptomycin (Pen Strep) (Gibco, Life Technologies, Carlsbad, CA, USA) and 10% fetal bovine serum (FBS) (Hyclone, Waltham, MA, USA). Cells were incubated at 37°C and 5% CO2 in a humidified incubator.

#### 2.1.2 RNAi-mediated transfection

Prostate cancer cells, PC3 and 22RV1, were seeded and transfected with BAT1 siRNA (si-BAT1) (Sigma, St. Louis, MO) using Lipofectamine RNAiMAX transfection reagent (Invitrogen, Carlsbad, CA) and OPTI-MEM Reduced Serum Medium (Life Technologies, Carlsbad, CA) following the manufacturer’s protocol. These cells were used for *in vitro* experiments for transient transfection.

The BAT1 siRNA sequence used was: sense 5’-CUUUCUCGGUAUCAGCAGUdTdT-3’ and anti-sense 5’-ACUGCUGAUACCGAGAAAGdTdT-3’. The control for this transfection was PC3 or 22RV1 cells transfected with the Mission Negative Universal Control siRNA sequence targeting no gene (Sigma, St. Louis, MO).

#### 2.1.3 BAT1 cDNA prostate cancer cells

Prostate cancer cells, PC3 and 22RV1, were seeded and transfected with BAT cDNA-GFP tagged lentiviral particles (BAT1cDNA) (pLenti-C-mGFP vector) (Origene, Rockville, MD, USA) using Polybrene transfection reagent (EMD Millipore, Burlington, MA) and OPTI-MEM Reduced Serum Medium. Cells were selected by culturing in the presence of puromycin. These cells were used for *in vitro* and *in vivo* experiments for stable cell line transfection. The control for this transfection was PC3 or 22RV1 with no transfection.

#### 2.1.4 BAT1 shRNA prostate cancer cells

Prostate cancer 22RV1 cells were seeded and transfected with BAT1 shRNA lentiviral particles (shBAT1) (pLKO.1 vector) (Clone ID: TRCN0000074383) (Sigma, St. Louis, MO) using Polybrene transfection reagent (EMD Millipore, Burlington, MA) and OPTI-MEM Reduced Serum Medium. Cells were selected by culturing in the presence of puromycin. These cells were used for *in vivo* experiments for stable cell line transfection. The control was mice that had 22RV1 cells with no transfection. The BAT1 shRNA lentiviral particle (pLKO.1 vector) sequence used was: CCGGCCTCAACCTCAAACACATTAACTCGAGTTAATGTGTTTGAGGTTGAGGTTTTT.

#### 2.1.5 Western blot

Cells were trypsinized, lysed using cell lysis buffer, and centrifuged. Protein concentration was determined using the Bio-Rad DC Protein Assay Kit and a spectrophotometer at 750 nm to obtain the quantity of protein in ug/uL. Forty (ug) of protein were loaded and separated using 12% SDS-PAGE and transferred onto a PVDF membrane (Bio-Rad, Hercules, CA, USA), followed by blocking in 5% BSA (Fisher Scientific, Fair Lawn, NJ, USA) for 1 hour at room temperature. BAT1 rabbit monoclonal antibody (1:1000 dilution) (Epitomics, Burlingame, CA) and anti-GFP (1:10000 dilution) (Abcam, Cambridge, MA, USA) were added and incubated overnight at 4 °C. Detection was achieved with the appropriate secondary antibody and enhanced chemiluminescence (ECL) kit (Bio-Rad, Hercules, CA, USA). Image J software (NIH, Bethesda, MD, USA) was used to quantitatively analyze the protein expression levels. β-actin (Sigma, A5441, Monoclonal Anti- β Actin Mouse) protein expression was used as the loading control.

#### 2.1.6 Migration assay

Cell migration was assayed using the wound healing method. Control PC3, siBAT1 PC3 or BAT1cDNA PC3 cells were seeded at a density of 2x10^5^ cells/mL and grown in a monolayer in six-well dishes until 95% confluent. Cells were serum starved in RPMI medium overnight. Cells were wounded in the center of the well using a 200 μL pipette tip, washed with 1X PBS, and incubated with RPMI-1640 serum-containing medium. Images of the wound were obtained at 0, 12, and 24 hours after wounding at a 4X magnification using a Nikon Eclipse TS100 microscope (Nikon, Tokyo, Japan). Wound width was measured using the Image Pro Plus Software (Meyer Instruments, Inc., Houston, TX, USA). 22RV1 cells were not used for the migration assay because these cells do not grow in a confluent monolayer.

#### 2.1.7 Invasion assay

Cell invasion assay was performed using the Boyden chamber method. siBAT1 or BAT1cDNA PC3 and 22RV1 cells were seeded at a density of 5x10^4^ cells/mL in serum-free RPMI-1640 medium in the upper chamber membrane of 24-well Transwell inserts (Corning, Corning, NY, USA) previously coated with laminin (Becton Dickinson, Franklin Lakes, NJ, USA). The lower chamber contained 600 μL of RPMI-1640 medium with 10% FBS and 5% Pen Strep. Cells were incubated at 37°C and 5% CO2 for 12 and 24 hours. Cells that did not invade the membrane from the insert were removed with a cotton swab dipped in 1X PBS. The membrane was then fixed by submerging the insert in 10% formalin (Thermo Scientific Waltham, MA, USA) and counterstained with hematoxylin (American Master Tech, Lodi, CA, USA). The Boyden membrane was removed and mounted on a glass slide. Images were obtained using 4X magnifications with a Nikon Eclipse TS100 microscope (Nikon, Tokyo, Japan). Invasive cells were counted using the Image Pro Plus Software (Meyer Instruments, Inc., Houston, TX, USA).

#### 2.1.8 Cell proliferation

PC3 and 22RV1 siBAT1 or BAT1cDNA cells were seeded at a density of 1x10^4^ cells/well in a 96-well plate. Cell proliferation was assayed at 24 hours using 20 µL of CellTiter 96^®^ AQueous One Solution Reagent (Promega, Madison, WI, USA) and 100 µL of RPMI medium, and incubated for 2 hours at 37°C and 5% CO2 in a humidified incubator. The plates were read at 490 nm using the xMarkTM Microplate Absorbance Spectrophotometer (Bio-Rad, Hercules, CA, USA).

#### 2.1.9 Cell viability

Cell viability and apoptosis assays were performed using flow cytometry (FACs analysis) with the Muse Cell Analyzer (EMD Millipore Merck KGaA, Darmstadt, Germany). PC3 and 22RV1 cells were seeded and grown in a 6-well cell culture plate, transfected with either siBAT1 or BAT1cDNA, and collected into tubes 24 hours after transfection. Cells were suspended in 1X PBS and 1 mL was transferred to a new tube. Twenty μL of cell suspension were stained with 380 μL of the Muse Count and Viability reagent and incubated (protected from light) for 5 minutes at room temperature. For 22RV1 cells, the protocol was the same with the exception that 20 μL of the Muse cell dispersal reagent (EMD Millipore Merck KGaA, Darmstadt, Germany) was added to 20 μL cells with 380 μL of the Muse Count and Viability reagent to each sample.

#### 2.1.10 Apoptosis

siBAT1 or BAT1cDNA PC3 cells (1x10^7^) were resuspended in 1X PBS, stained with 100 μL of annexin-V, and incubated and protected from light for 20 minutes at room temperature. For 22RV1 cells, 2x10^5^-2x10^7^ cells/1mL was added to tubes, 50 µL of cell suspension was mixed with 50 µL of the Muse cell dispersal reagent and were incubated protected from light for 20 minutes at room temperature. Cell populations were gated using the Muse software.

#### 2.1.11 RNA isolation and qRT-PCR

Total RNA was extracted from siBAT1 or BAT1cDNA PC3 and 22RV1 cells using the RNeasy Mini Isolation Kit (Qiagen, Venlo, The Netherlands) following the manufacturer’s protocol. cDNA was synthesized using the iScript cDNA Synthesis Kit (Bio-Rad, USA) according to the manufacturer’s instructions. PCR amplification was done using real-time PCR with the iQ SYBR Green Supermix (Bio-Rad, USA) and the Step One Plus Real-time PCR System (Applied Biosystems, Carlsbad; CA, USA) as follows: 95 °C for 5 min, 95 °C for 15 seconds and 60 °C for 1 min at 40 cycles. Changes in mRNA expression were analyzed using the ΔΔCt method and the Step One Software. Negative fold changes were determined by dividing 1/Average Fold Change obtained. Expression levels were normalized to GAPDH expression.

### 2.2 *In vivo* experiments

#### 2.2.1 Orthotopic mouse model

For *in vivo* experiments, human prostate cancer 22RV1 cells (250,000 cells contained in 70 μL) transfected with shBAT1 (n=5 mice) or BAT1cDNA (n=5 mice) were mixed with 30 uL collagen and injected into the anterior prostate lobules of 7–8-week-old male ICR-SCID mice (Taconic, Germantown, NY, USA) to generate 2 tumors (one per each prostate lobule). Our control mice (n=6) were injected with non-transfected 22RV1 cells. Mice were kept in a pathogen-free environment under the Institutional Animal Care and Use Committee regulations at The University of Puerto Rico Medical Sciences Campus animal facility (Protocol #A8700110). Mice were anesthetized and sacrificed. Furthermore, prostate tumors and livers were collected, weighed, and used for histologic analysis.

#### 2.2.2 Tissue collection and histological examination

Tumors collected were fixed in 10% buffered formalin and embedded in paraffin. Formalin-fixed paraffin-embedded (FFPE) tumors were cut at 5 µm sections using a microtome (Leica Microsystems, Wetzlar, Germany) and mounted on slides. Slides were deparaffinized in xylene, hydrated using serial descending concentrations of alcohol, stained with hematoxylin, followed by stain differentiation with eosin, and dehydrated with increasing serial dilutions of ethanol and xylene. Slides were mounted with coverslips using permount-mounting medium. As described by Lsaacs and Hukku (33) tumors were classified into four categories by a degree of differentiation: well differentiated, moderately differentiated, poorly differentiated, and anaplastic. Well-differentiated tumors are characterized by the presence of glandular structures, lumen, basement membrane, and stroma. Moderately differentiated tumors are characterized by smaller glandular structures with the lumen obstructed by tumor cells. However, the basement membrane and stroma remained intact. Tumors classified as poorly differentiated have an absence of glandular structures, and basement membrane, and do not show a consistent relationship between tumor cells and stroma. Individual tumor cells, however, still show a normal nucleus to cytoplasm ratio. Tumors classified as anaplastic lack appearance of tissue organization and individual tumor cells show irregular nucleus size and abnormal nucleus to cytoplasm ratio. Our tumors collected represent n=6 tumors for control, n=5 tumors for BAT1cDNA, and n=3 tumors for shBAT1.

#### 2.2.3 Immunohistochemistry

Formalin-fixed paraffin-embedded (FFPE) mice tumor tissues were dewaxed in xylene and rehydrated in descending concentrations of alcohol and deionized water. Antigen retrieval was performed using the Antigen Unmasking Solution (Vector Laboratories, Burlingame, CA, USA) followed by quenching of endogenous peroxidase with 3% v/v H_2_O_2_. Sections were blocked for 1 hour with horse serum (R.T.U Vectastain Kit, Vector Laboratories, Burlingame, CA, USA) and left overnight with the primary antibody at 4°C in a humidified chamber. The primary antibodies used were: BAT1 (1:50 dilution) (Epitomics, Burlingame, CA, USA), Cleaved Caspase-3 (1:200 dilution) (Cell Signaling, Danvers, MA, USA), Ki67 (1:1000 dilution) (Vector Laboratories, Burlingame, CA, USA), TNF-α (1:100 dilution) (Abcam, Cambridge, MA, USA), IL-6 (1:25 dilution) (Novus Biologicals, Littleton, CO, USA), and MMP10 (1:100 dilution) (Abcam, Cambridge, MA, USA). Protein expression was detected with the peroxidase substrate kit (ImmPACT DAB) (Vector Laboratories, Burlingame, CA, USA). Hematoxylin was used as a counterstain. Digital images were obtained using an Olympus IX71 Inverted microscope (Olympus America, Melville, NY, USA) at a 20x magnification. To quantify BAT1, Cleaved Caspase-3 expression, and Ki67 expression, a set of 5 random fields per slide were chosen and the number of total cells, negative cells, and positive cells were quantified. The number of positive cells in response to the primary antibody was divided over the number of total cells and a percentage per field was determined. The 5 total fields per slide were then averaged to generate a percentage of positive cells. To quantify MMP10, TNF-α, and IL-6 expression, a subjective scale from 1-4 was used. In this scale, we gave a score of one (1) if 25% or less of the tumor cells were stained, a score of two (2) if 26% to 50% of the tumor cells were stained, a score of three (3) if 51% to 75% of the tumor cells were stained, and a score of four (4) if more than 75% of the tumor cells were stained. The score was given in a blind manner. All images were analyzed using Image the Pro Plus Software (Meyer Instruments, Inc., Houston, TX, USA).

#### 2.2.4 Immunofluorescence

Paraffin-embedded tissue (FFPE) was dewaxed in xylene (x2) and rehydrated in descending concentrations of alcohol and deionized water. Antigen retrieval was performed using the Antigen Unmasking Solution (Vector Laboratories, Burlingame, CA, USA) and the heat was applied using a microwave. Slides were placed on ice and then followed by quenching of endogenous peroxidase with 3% v/v H_2_O_2_. Sections were blocked for 1 hour with 10% FBS and left overnight with the primary antibody at 4°C in a humidified chamber. The primary antibody used was CD31 (1:50 dilution) (Abcam, Cambridge; MA, USA). The secondary antibody used was Alexa-Fluor 594 (anti-rabbit) 1:1000 (Molecular Probes, Life Technologies, Carlsbad, CA, USA) and, nuclei were stained with DAPI 1:5000 (Santa Cruz Biotechnology, Santa Cruz, CA, USA). Digital images were obtained using an Olympus IX71 Inverted microscope (Olympus America, Melville, NY, USA) at a 20X magnification. To quantify CD31, sets of 5 random fields were chosen per slide and the total number of blood vessels was counted and averaged per slide.

#### 2.2.5 Statistical analysis

All *in vitro* experiments were performed in triplicates (n=3). Results represent the mean ± standard error of the mean (SEM). Differences between treatments were analyzed using the Student’s t-test at a 95% confidence interval. P-values <0.05 were considered statistically significant. Statistical analysis was done using GraphPad Prism Software (GraphPad Software, CA, USA). For *in vivo* statistical analysis was done using the analysis of variance (ANOVA) and the GraphPad Prism Software (GraphPad Software, CA, USA).

## 3 Results

### 3.1 BAT1 expression was decreased after siRNA transfection and increased after cDNA transfection in PC3 and 22RV1 cells

**Figure 1:**
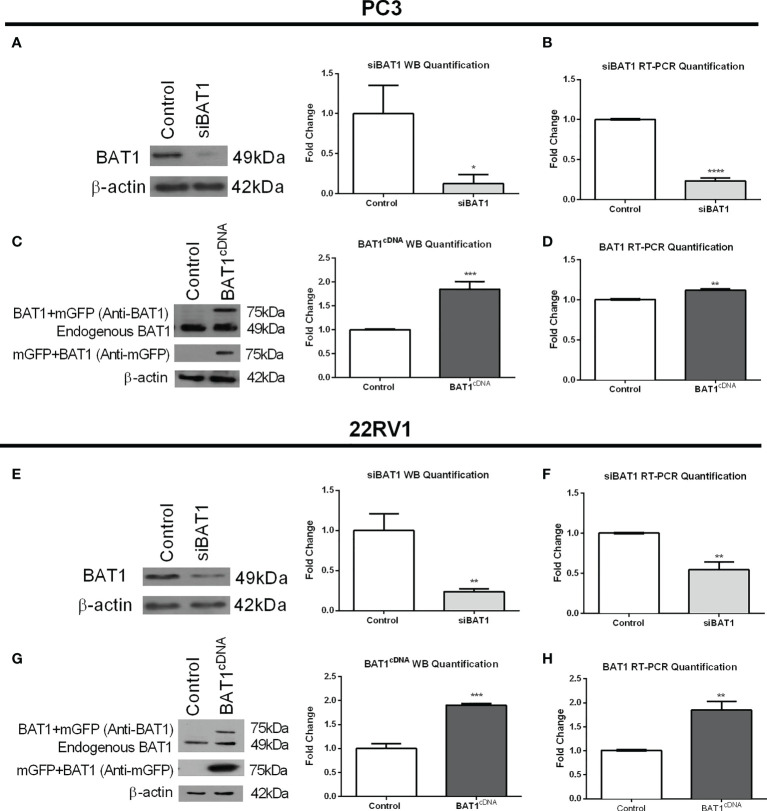
BAT1 Expression was decreased after siRNA Transfection and increased after cDNA Transfection in PC3 and 22RV1 cells. The samples were collected for a period of 24 hours. **(A)** Representative images and quantification of BAT1 protein expression in PC3 prostate cancer cells using western blot analysis showed a significant decrease in siBAT1 transfected cells when compared to control (*P<0.05). **(B)** Quantification of BAT1 RNA expression using RT-PCR in PC3 prostate cancer cells showed a significant decrease in BAT1 expression in siBAT1 transfected cells when compared to control (****P < 0.0001). **(C)** Representative images and quantification of BAT1 protein expression in PC3 prostate cancer cells using western blot analysis showed a significant increase in protein expression in BAT1cDNA when compared to control (***P < 0.001). **(D)** Quantification of BAT1 RNA expression using RT-PCR in PC3 prostate cancer cells showed a significant increase in BAT1 protein expression in BAT1cDNA cells when compared to control (**P < 0.01). **(E)** Representative images and quantification of BAT1 protein expression in 22RV1 prostate cancer cells using western blot analysis showed a significant decrease in siBAT1 transfected cells when compared to control (**P < 0.01). **(F)** Quantification of BAT1 RNA expression using RT-PCR in 22RV1 prostate cancer cells showed a significant decrease in BAT1 expression in siBAT1 transfected cells when compared to control (**P < 0.01) **(G)** Representative images and quantification of BAT1 protein expression in 22RV1 prostate cancer cells using western blot analysis showed a significant increase in BAT1cDNA when compared to control (***P < 0.001). **(H)** Quantification of BAT1 RNA expression using RT-PCR in 22RV1 prostate cancer cells showed a significant increase in BAT1 protein expression in BAT1cDNA cells when compared to control (**P < 0.01).

To investigate the role of BAT1 in prostate cancer cells, we transfected PC3 and 22RV1 cells with siBAT1 to down-regulate BAT1 expression or BAT1cDNA to overexpress BAT1 expression. Protein and RNA extraction from transfected cells were obtained and subjected to SDS-PAGE western blot analysis or qRT-PCR to determine efficient transfection. siBAT1 PC3 cells showed significantly less expression of BAT1 by western blot analysis and qRT-PCR when compared to control (**Figures 1A, B**). Additionally, siBAT1 22RV1 cells showed significantly less expression of BAT1 by western blot analysis and qRT-PCR when compared to control (**Figures 1E, F**). In contrast, BAT1cDNA PC3 and 22RV1 cells showed a significant increase in BAT1 expression by western blot and qRT-PCR analysis when compared to control (**Figures 1C, D, G, H**).

### 3.2 BAT1 down-regulation significantly increased cell migration and BAT1 overexpression significantly decreased cell migration in PC3 cells

**Figure 2:**
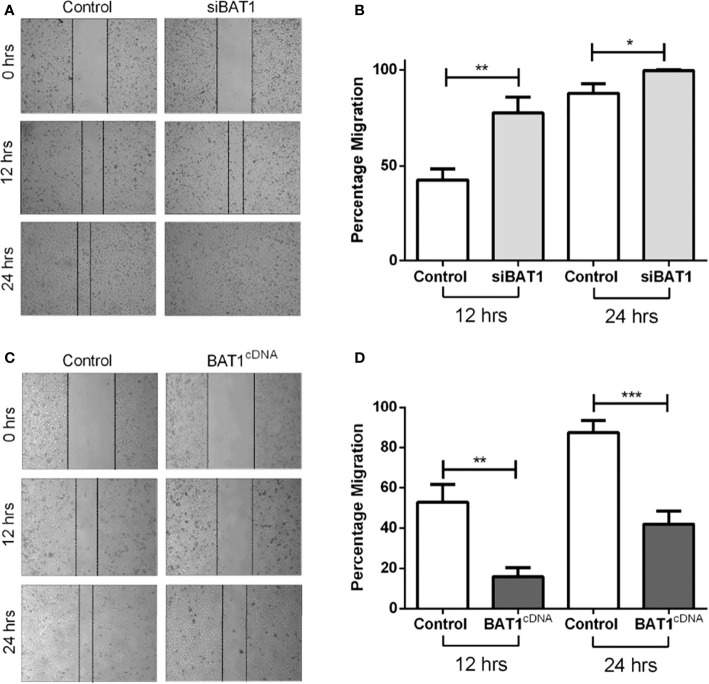
BAT1 down-regulation increased PC3 prostate cancer cell migration and BAT1 overexpression decreased PC3 prostate cancer cell migration. **(A)** Representative images of Control PC3 and siBAT1 PC3 cells using 4X magnification. **(B)** Relative invasion of siBAT1 PC3 cells caused a significant increase in migration at 12hrs (**P < 0.01) and 24hrs (*P < 0.05) when compared to control. **(C)** Representative images of Control PC3 and BAT1cDNA PC3 cells using 4X magnification. **(D)** Relative invasion of (+) BAT1cDNA PC3 cells caused a significant decrease in migration at 12hrs (**P < 0.01) and 24hrs (***P < 0.001) when compared to control.

The ability of PC3 cells to migrate after BAT1 expression was altered. Results showed that siBAT1 PC3 cells had a significant increase in migratory potential at 12 hours and at 24 hours when compared to control (**Figures 2A, B**). Conversely, BAT1cDNA PC3 cells showed a significant decrease in migratory potential of 37% at 12 hours and 44% at 24 hours when compared to control (**Figures 2C, D**). 22RV1 cells were not used for the migration assay because these cells do not grow in a confluent monolayer.

### 3.3 BAT1 down-regulation significantly increased cell invasion and BAT1 overexpression significantly decreased cell invasion in PC3 and 22RV1 cells

**Figure 3:**
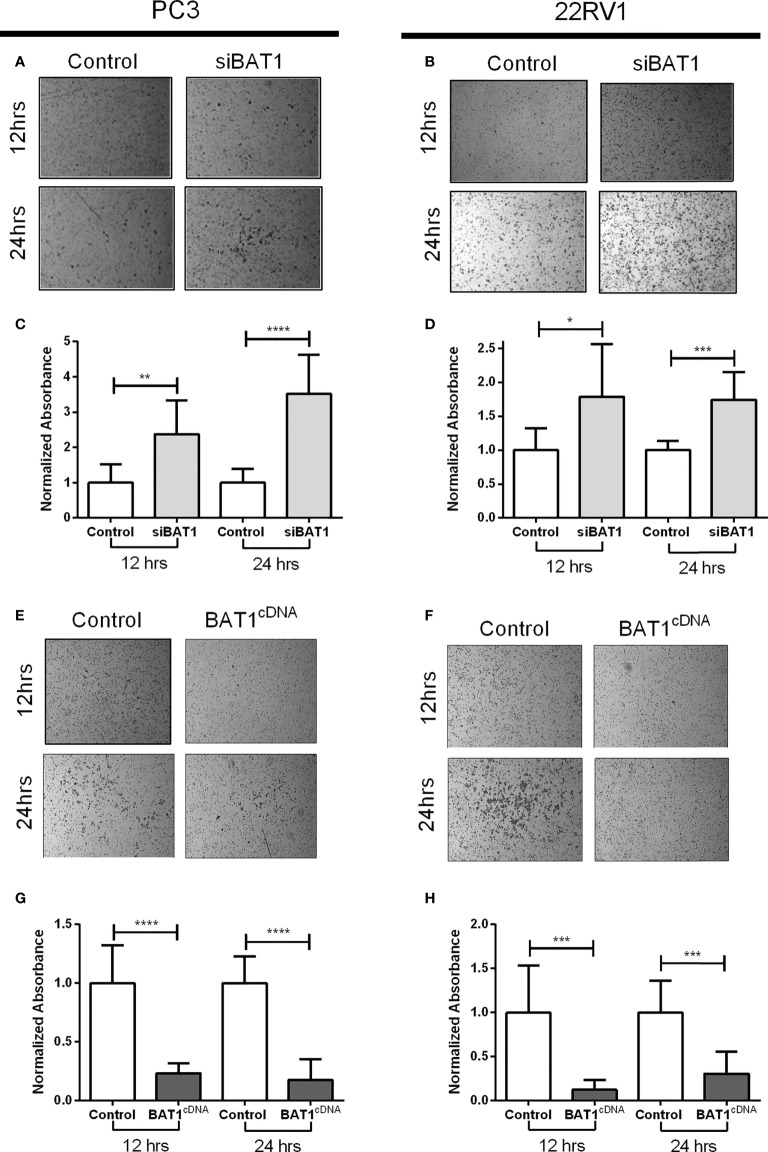
BAT1 down-regulation increased PC3 and 22RV1 prostate cancer cell and BAT1 overexpression decreased PC3 and 22RV1 prostate cancer cell invasion. **(A, B)** Representative images of invasive siBAT1 PC3 and 22RV1 cells at a 4X magnification. **(C)** Relative invasion of siBAT1 PC3 cells caused a significant increase in invasion when compared to control at 12hrs (**P < 0.01) and 24hrs (****P < 0.0001). **(D)** Relative invasion of siBAT1 22RV1 cells caused a significant increase in invasion when compared to control at 12hrs (*P < 0.05) and 24hrs (***P < 0.001). **(E, F)** Representative images of invasive BAT1cDNA PC3 and 22RV1 cells at a 4X magnification. **(G)** Relative invasion of BAT1cDNA PC3 cells caused a significant decrease in invasion when compared to control at 12hrs (****P < 0.0001) and 24hrs (****P < 0.0001). **(H)** Relative invasion of BAT1cDNA 22RV1 cells caused a significant decrease in invasion when compared to control at 12hrs (***P < 0.001) and 24hrs (***P < 0.001).

The effects of BAT1 expression in invasion were examined using the Transwell assay siBAT1 PC3 cells significantly increased cell invasion at 12 hours and 24 hours when compared to control (**Figures 3A, C**). Moreover, siBAT1 22RV1 cells significantly increased cell invasion at 12 hours and 24 hours when compared to control (**Figures 3B, D**). On the contrary, BAT1cDNA PC3 cells significantly decreased cell invasion at 12 hours and 24 hours when compared to control (**Figures 3E, G**). Furthermore, cell invasion significantly decreased in BAT1cDNA 22RV1 cells at 12 hours and 24 hours when compared to control (**Figures 3F, H**). Also, to determine if changes in migration and invasion were due to changes in proliferation and apoptosis, we performed a proliferation assay using MTT and an apoptosis assay using FACs analysis. Results showed no significant changes in these molecular hallmarks of cancer (**Supplemental Figures S1–S3**). These data suggest that BAT1 suppresses cell migration and invasion without altering proliferation or apoptosis *in vitro*.

### 3.4 Alteration of BAT1 expression showed changes in genes associated with inflammation, adhesion, and metastasis in PC3 and 22RV1 cells using qRT-PCR

**Figure 4:**
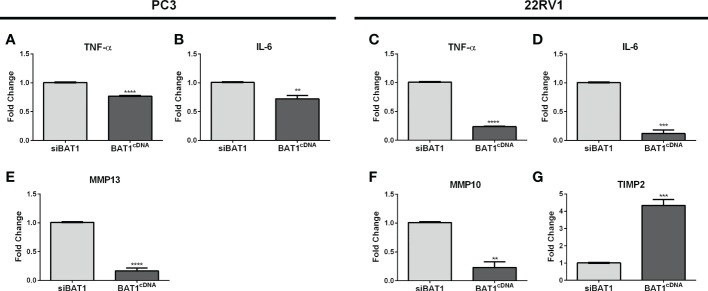
BAT1 expression showed changes in genes associated with inflammation, adhesion, and metastasis in PC3 and 22RV1 cells using qRT-PCR. The samples were collected for a period of 24 hours. **(A, C)** BAT1cDNA PC3 and 22RV1 cells showed a significant decrease in TNF-α expression when compared to siBAT1 (****P < 0.0001). **(B, D)** BAT1cDNA PC3 and 22RV1 cells showed a significant decrease in IL-6 expression when compared to siBAT1 (**P < 0.01) (***P < 0.001). **(E)** BAT1cDNA PC3 cells showed a significant decrease in MMP13 expression when compared to siBAT1 (****P < 0.0001). **(F)** BAT1cDNA 22RV1 cells showed a significant decrease in MMP10 expression when compared to siBAT1 (**P < 0.01). **(G)** BAT1cDNA 22RV1 cells showed a significant increase in TIMP2 expression when compared to siBAT1 (***P < 0.001).

Previous studies have associated the role of BAT1 as an anti-inflammatory gene in diseases such as Chagas cardiomyopathy and *Plasmodium vivax* malaria through the modulation of tumor necrosis factor-alpha (TNF-α) and interleukin-6 (IL-6) (16, 18). It is known that inflammation can eventually lead to cell migration and metastasis (34, 35). Thus, we wanted to identify a detailed signaling pathway that might be involved with BAT1 expression in PCa cell progression using qRT-PCR. Results demonstrated significant changes in TNF-α, IL-6, MMP-10, and TIMP2 expression. BAT1cDNA PC3 cells significantly decreased TNF-α, IL-6, and matrix metallopeptidase 13 (MMP-13) expression when compared to siBAT1 PC3 cells (**Figure 4** and **Table 1**). Results showed significant decreases in TNF-α, IL-6, and matrix metallopeptidase 10 (MMP-10) expressions in BAT1cDNA 22RV1 cells when compared to siBAT1 22RV1 cells (**Figures 4C, D, F**). Additionally, tissue inhibitor of metalloproteinase 2 (TIMP2) expression, was significantly increased in BAT1cDNA 22RV1 cells when compared to siBAT1 22RV1 cells. These results suggest that BAT1 is altering inflammatory cytokine expression and metastatic gene expression.

**Table 1:** Alteration of BAT1 expression showed changes in genes associated with inflammation, adhesion and metastasis in PC3 and 22RV1 cells using qRT-PCR.

Cell Line Transfected	Gene	Function	Fold Change
PC3 BAT1^cDNA^ vs PC3 siBAT1	TNF-α	Involved in systemic inflammation	-1.35
PC3 BAT1^cDNA^ vs PC3 siBAT1	IL-6	Pro-inflammatory cytokine	-0.92
PC3 BAT1^cDNA^ vs PC3 siBAT1	MMP13	Involved in cancer migration	-6.25
22RV1 BAT1^cDNA^ vs 22RV1 siBAT1	TNF-α	Involved in systemic inflammation	-4.34
22RV1 BAT1^cDNA^ vs 22RV1 siBAT1	IL-6	Pro-inflammatory cytokine	-9.09
22RV1 BAT1^cDNA^ vs 22RV1 siBAT1	TIMP2	Directly suppress the proliferation of endothelial cells	4.33
22RV1 BAT1^cDNA^ vs 22RV1 siBAT1	MMP10	Involved in cancer migration	-4.00

### 3.5 *In vivo* expression of BAT1, Ki-67, TNK- α, IL-6 and MMP10

**Figure 5:**
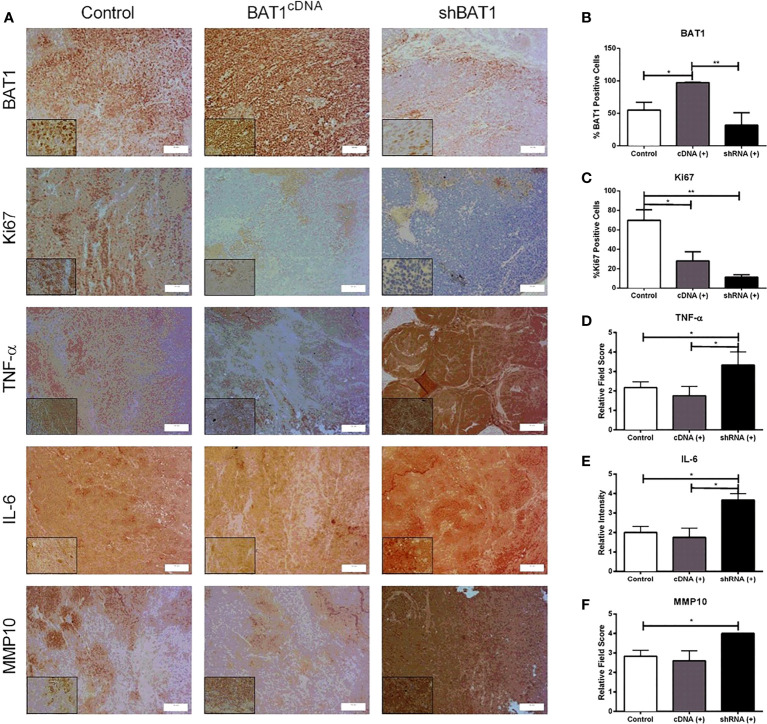
*In vivo* expression of BAT1, Ki67, TNF-α, IL-6, and MMP10. All of the samples were collected for a period of 24 hours. **(A)** Representative images of immunohistochemical staining BAT1 expression in mice prostate tumors previously injected with 22RV1 cells, BAT1cDNA 22RV1 cells, or shBAT1 22RV1 cells at a 20X (50μm) or 60X magnification (10μm) magnification. **(B)** Quantification mice prostate tumors obtained from BAT1cDNA mice prostate tumors showed a significant increase in BAT1 expression using immunohistochemistry when compared to control (*P < 0.05) and shBAT1 (**P < 0.01). **(C)** shBAT1 (**P < 0.01) and BAT1cDNA (*P < 0.05) tumors showed a significant decrease in Ki67 expression when compared to control. **(D)** shBAT1 tumors showed a significant increase in TNF-α expression when compared to control and BAT1cDNA (*P < 0.05). **(E)** shBAT1 tumors showed a significant increase in IL-6 expression when compared to control and BAT1cDNA mice prostate tumors (*P < 0.05). **(F)** shBAT1 tumors showed a significant increase in MMP10 expression when compared to control (*P < 0.05).

Prior to 22RV1 prostate cancer cells injection into SCID mice prostate lobules, we confirmed transfection of BAT1 expression using western blot analysis (**Supplemental Figure S4**). PC3 cells were not used for *in vivo* experiments because they do not grow properly in these models. Tumors developed in SCID mice were examined by immunohistochemistry. H&E sections were evaluated by a pathologist for the observation of inflammation and histological classification. The tumors were classified as poorly differentiated or anaplastic. (**Supplemental Figure S5**). BAT1 expression was significantly increased in BAT1cDNA prostate tumors when compared to control and shBAT1 prostate tumors (**Figures 5A, B**). Although tumors from shBAT1 transfected mice showed no statistical significance in BAT1 expression when compared to control, results represent a tendency to decrease expression.

To evaluate whether BAT1 expression altered proliferation, apoptosis, or angiogenesis *in vivo*, mice prostate tumor sections were examined by immunohistochemistry or immunofluorescence. The nuclear marker Ki67 was used to determine changes in proliferation. Mice prostate tumors developed from BAT1cDNA and shBAT1 transfected cells significantly decreased cell proliferation when compared to control (**Figure 5C**). shBAT1 tumors showed an increase in TNF-α expression when compared to control and BAT1cDNA (**Figure 5D**). Apoptosis using the pro-apoptotic marker cleaved caspase-3 showed no change in expression for all groups (**Supplemental Figure S6**). To determine if BAT1 expression plays a role in angiogenesis, mice prostate tumor sections were subjected to immunofluorescence using the angiogenesis marker CD31. Results showed no significant change in the number of blood vessels for all groups (**Supplemental Figure S7**).

**Figure 6:**
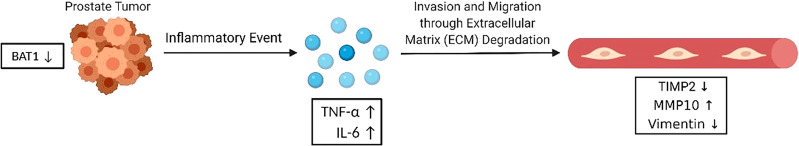
BAT1 promotes *in vitro* invasion and migration. BAT1 down-regulation leads to activation of pro-inflammatory cytokines TNF-α and IL-6, which leads to the secretion of MMP-10 and inhibition of TIMP2. MMP-10 may degrade the ECM and promote PCa invasion and migration through extracellular matrix degradation.

*In vitro* results showed changes in cell invasion, migration, and genes associated with inflammation and metastasis. In an inflammatory event, the downregulation of BAT1 may stimulate increases in pro-inflammatory cytokines, TNF-α and IL-6. Cells may lose their ability to maintain tight junctions and cell-cell adhesion leading to invasive potential through the secretion of MMPs and the inhibition of TIMPs. MMPs degrade the extracellular matrix leading to invasion of mesenchymal cells into the bloodstream, thus promoting metastasis. Based on this signaling pathway, we identified significant changes in TNF-α, IL-6, and matrix metallopeptidase 10 (MMP-10) gene expression when we performed RT-PCR in PC3 and 22RV1 prostate cancer cells, thus, we wanted to determine if there would be significant changes in expression in an *in vivo* mouse model. Immunohistochemistry analysis in mice prostate tumors showed that shBAT1 significantly increased TNF-α and IL-6 expression when compared to control and BAT1cDNA tumors (**Figures 5D, E**). Additionally, MMP-10 expression was significantly increased in shBAT1 mice prostate tumors when compared to control (**Figure 5F**). These findings suggest that BAT1 down-regulation leads to activation of pro-inflammatory cytokines TNF-α and IL-6, which leads to the secretion of MMP-10, inhibition of TIMP2, and promotion of invasion and migration (**Figure 6**).

## 4 Discussion

Studies have shown that unidentified MHC class III genes play a role in inflammatory and immunopathological responses in diseases such as ulcerative colitis, diabetes, rheumatoid arthritis, and multiple sclerosis (5–12). The RNA helicase and MHC class III gene, BAT1, has been identified as an anti-inflammatory gene in diseases such as Chagas cardiomyopathy, Plasmodium vivax malaria, multiple sclerosis, and insulin dependent diabetes mellitus by the modulation of the pro-inflammatory cytokines TNF-α, IL-6 and IL-1 expression (12–14, 36). Nevertheless, the functional role of BAT1 in PCa recurrence has not been revealed. Immunohistochemical analysis demonstrated that BAT1 expression was differentially expressed in patients with high Gleason scores when compared to PCa patients with low Gleason scores. In this work, we determined the role of BAT1 in migration, invasion, and gene expression using *in vitro* and *in vivo* PCa models.

In this study, genetic down-regulation of BAT1 significantly increased cell migration, invasion, and expression of TNF-α, IL-6, MMP10, and MMP13 while it decreased the expression of TIMP2. Conversely, overexpression of BAT1 significantly decreased cell migration, invasion, and expression of TNF-α, IL-6, MMP10, and MMP13 while it increased the expression of TIMP2. However, apoptosis and cell proliferation were not affected in either group due to no significant changes observed in the proliferation and apoptosis assays using FACs analysis. The results suggest that BAT1 mechanisms do not involve changes in proliferation or apoptotic pathways. Taken together, these data indicate that BAT1 overexpression may function as an anti-inflammatory gene and suppressor of metastasis and invasion in PCa. Since metastasis and invasion eventually may promote recurrence, understanding and identifying the mechanisms by which BAT1 functions may lead to better treatment options for recurrent PCa patients.

Cancer cell invasion is an important hallmark of cancer and an essential step toward metastasis (37, 38). In our study, we observed increases in cell migration and invasion in PCa cells transfected with siBAT1 and decreases in cell migration and invasion in PCa cells transfected with BAT1cDNA. Based on these results, we wanted to determine changes in genes that were associated with invasion, adhesion, and metastasis. We expected to detect changes in matrix metalloproteinases (MMPs) and its inhibitor, tissue inhibitors of matrix metalloproteinases (TIMPs). Significant changes were determined in MMP-10, MMP-13, and TIMP2. These results were expected due to previously published data that have identified MMPs to be involved in EMT, which is related to the invasion and metastasis ability of tumors in various types of cancer, including PCa.

BAT1 expression has been implicated in alterations of inflammation, specifically, through the modulation of TNF-α and IL-6 expression in inflammatory diseases (15–18). In a tumor microenvironment or inflammatory event, mesenchymal cells can secrete TNF-α and IL-6 (32, 39). The pro-inflammatory cytokine TNF-α can subsequently lead to the release of MMPs (32). To study the functional role of BAT1 in inflammatory cytokine secretion, qRT-PCR was performed to confirm mRNA expressions of TNF-α and IL-6 *in vitro*. Results showed decreases in TNF-α and IL-6 expression in PCa cells treated with BAT1cDNA when compared to siBAT1. These results were expected due to studies identifying BAT1 polymorphisms (decreases in BAT1 expression) with inflammation in patients that had an immune response disease. These findings also may explain why we found changes in migratory and invasive potential. We subsequently measured changes in cell migration and invasion *in vitro*, which may be contributed to the expression of TNF-α and IL-6 in PC3 and 22RV1 cells. Based on these results, we hypothesized that the secretion of pro-inflammatory cytokines may lead to metastatic potential through EMT. To verify our hypothesis, Western Blot analysis was performed to confirm the overexpression of Vimentin and N-Cadherin proteins. However, results showed no significant changes in Vimentin and N-cadherin protein expression in siBAT1 PCa cells compared to control cells (**Figure S9**).

IHC analysis showed that shBAT1 mice prostate tumors increased TNF-α, IL-6, and MMP-10 expression. These findings correlated to our qRT-PCR results demonstrating an increase in MMP-10 expression *in vitro*. Additionally, MMP-10 (stromelysin-2) has been overexpressed in various cancers including gastric, bladder, renal, esophageal, skin, and non-small cell lung cancer (40, 41). MMP-10 plays an important role in the development and progression of malignant tumors. MMP-10 expression has been implicated in the modulation of invasion, apoptosis, angiogenesis, and cell proliferation in cancer (42).

This study has some limitations including that no migration and invasion studies were performed in the *in vivo* models, and additional experiments need to be performed to supplement and conclude that the promotion of invasion and migration is present in the *in vivo* model possibly due to the changes described on TNF- α, IL-6, MMP-10, and TIMP2 in this manuscript. Another limitation was that the migration assays *in vitro* were only performed using the PC3 cell line because the 22RV1 cell line grows in clusters and are not appropriate for the wound healing test. Lastly, for the *in vivo* experiments, only the 22RV1 cell line was used because previous studies in our laboratory had shown that they grow better in mice in comparison with the PC3 cell line.

## Data availability statement

The original contributions presented in the study are included in the article/**Supplementary Materia**l. Further inquiries can be directed to the corresponding author.

## Ethics statement

All animal experiments were performed by a protocol approved by the Institutional Animal Care and Use Committee (IACUC) at the University of Puerto Rico, Medical Sciences Campus.

## Author contributions

Conceptualization, MM-F and MS-V. Methodology: MM-F, OV, MS-V, MG-M. Software: AG-V, YR-R, DA-V, MS-V, AR-T, AR-M, VC-B, DN. Validation: MM-F. and MS-V. Formal analysis: MM-F, OV, and MS-V. Investigation: AG-V, YR-R, DA-V, MS-V, AR-T, AR-M, VC-B, DN. Resources: MM-F, MG-M, and MS-V. Data curation: MM-F and MS-V. Writing—original draft preparation: A G-V, YR-R, DA-V. Writing—review and editing: AG-V, YR-R, DA-V, MS-V, AR-T, AR-M, VC-B, DN, OV, MG-M. Visualization: MM-F and MS-V. Supervision: MM-F and MS-V. Project administration: MM-F. Funding acquisition: MM-F. All authors contributed to the article and approved the submitted version.

## Funding

This research was funded by Institutional funds from the University of Puerto Rico Comprehensive Cancer Center, the NIGMS-RISE Program (Grant Number #R25GM061838), the RCMI Pilot Project (Grant Number #8G12MD007600 and NIMHD: U54-MD007600), and the National Center Institute of the National Institutes of Health (Award Grant Number # U54CA096297/CA096300).

## Conflict of interest

The authors declare that the research was conducted in the absence of any commercial or financial relationships that could be construed as a potential conflict of interest.

## Publisher’s note

All claims expressed in this article are solely those of the authors and do not necessarily represent those of their affiliated organizations, or those of the publisher, the editors and the reviewers. Any product that may be evaluated in this article, or claim that may be made by its manufacturer, is not guaranteed or endorsed by the publisher.
