# Supplementary material for: HLA-BAT1 alters migration, invasion and pro-inflammatory cytokines in prostate cancer
Source: Front Oncol. 2022 Nov 23;12:969396. doi: 10.3389/fonc.2022.969396 (PMC9727259; doi:10.3389/fonc.2022.969396)
Supplement: Supplementary file 1 [file DataSheet_1.pdf]

Supplementary Material

1 Supplementary Figures and Tables

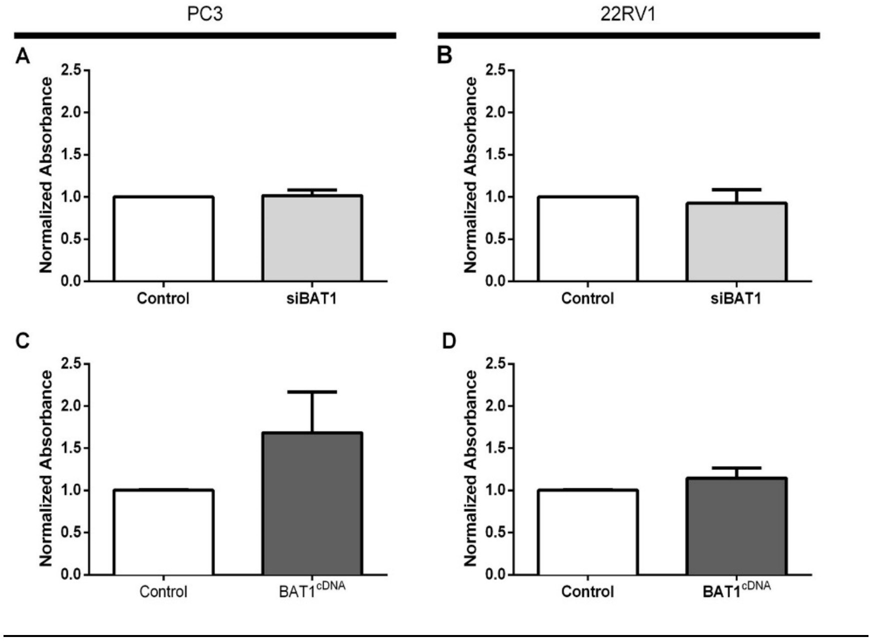

Supplementary Figure 1. BAT1 down-regulation or overexpression showed no change in cell proliferation in PC3 and 22RV1 cells. (A)(C) PC3 cells transfected with siBAT1 or BAT1cDNA showed no significant change in cell proliferation using MTS reagent when compared to control (P=0.6803) (P=0.2318). (B)(D) 22RV1 cells transfected with siBAT1 or BAT1cDNA showed no significant change in cell proliferation using MTS reagent when compared to control (P=0.4772) (P=0.2996).

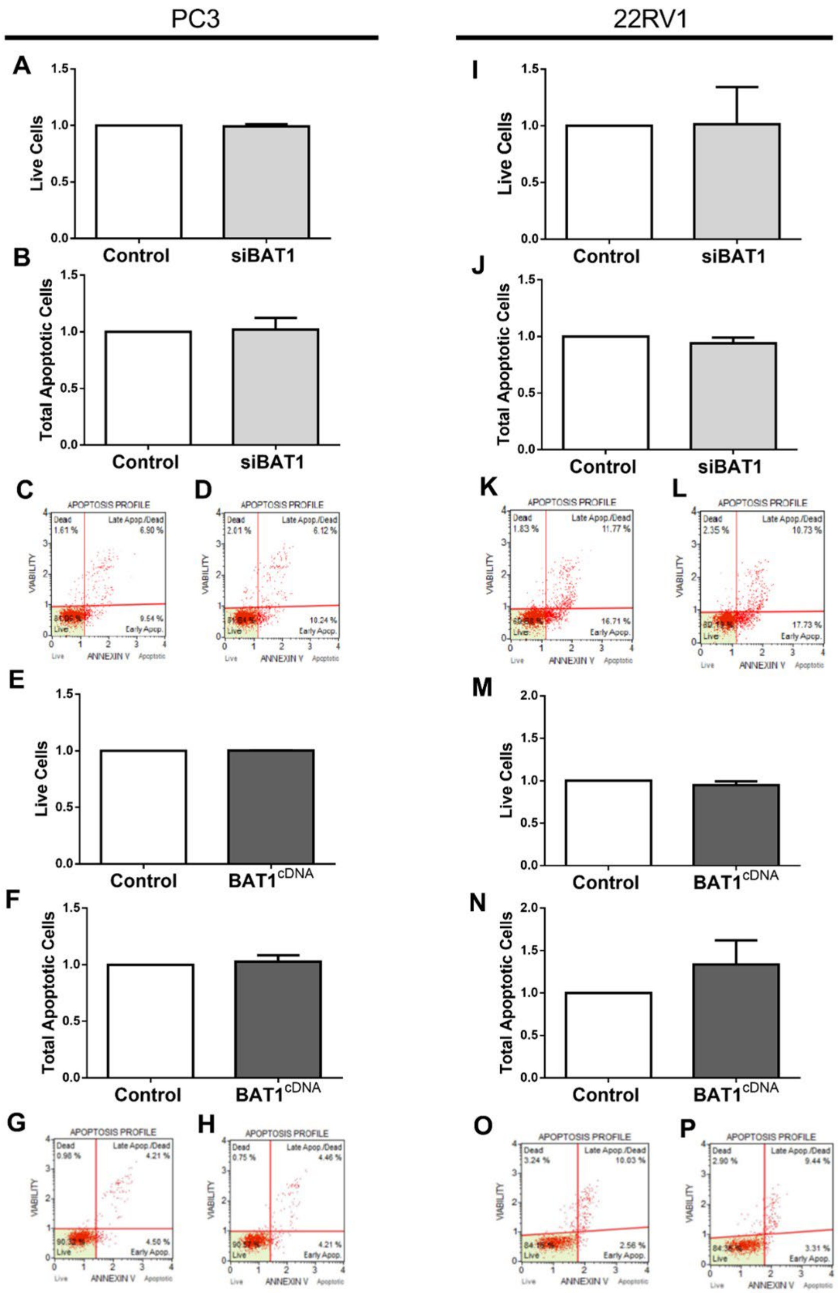

Supplementary Figure 2. BAT1 down-regulation or overexpression showed no change in apoptosis in PC3 and 22RV1 cells (A)(E) PC3 cells transfected with siBAT1 or BAT1cDNA showed no significant change in the percentage of live cells when compared to control. (B)(F) PC3 cells transfected with siBAT1 or BAT1cDNA showed no change in apoptosis when compared to control. (C)(G) Representative image of control PC3 cells using FACs analysis. (D)(H) Representative image of siBAT1 or BAT1cDNA cells using FACs analysis. (I)(M) 22RV1 cells transfected with siBAT1 or BAT1cDNA showed no significant change in the percentage of live cells when compared to control. (J)(N) 22RV1 cells transfected with siBAT1 or BAT1cDNA showed no change in apoptosis when compared to control. (K)(O) Representative image of control 22RV1 cells using FACs analysis. (L)(P) Representative image of siBAT1 or BAT1cDNA 22RV1 cells using FACs analysis.

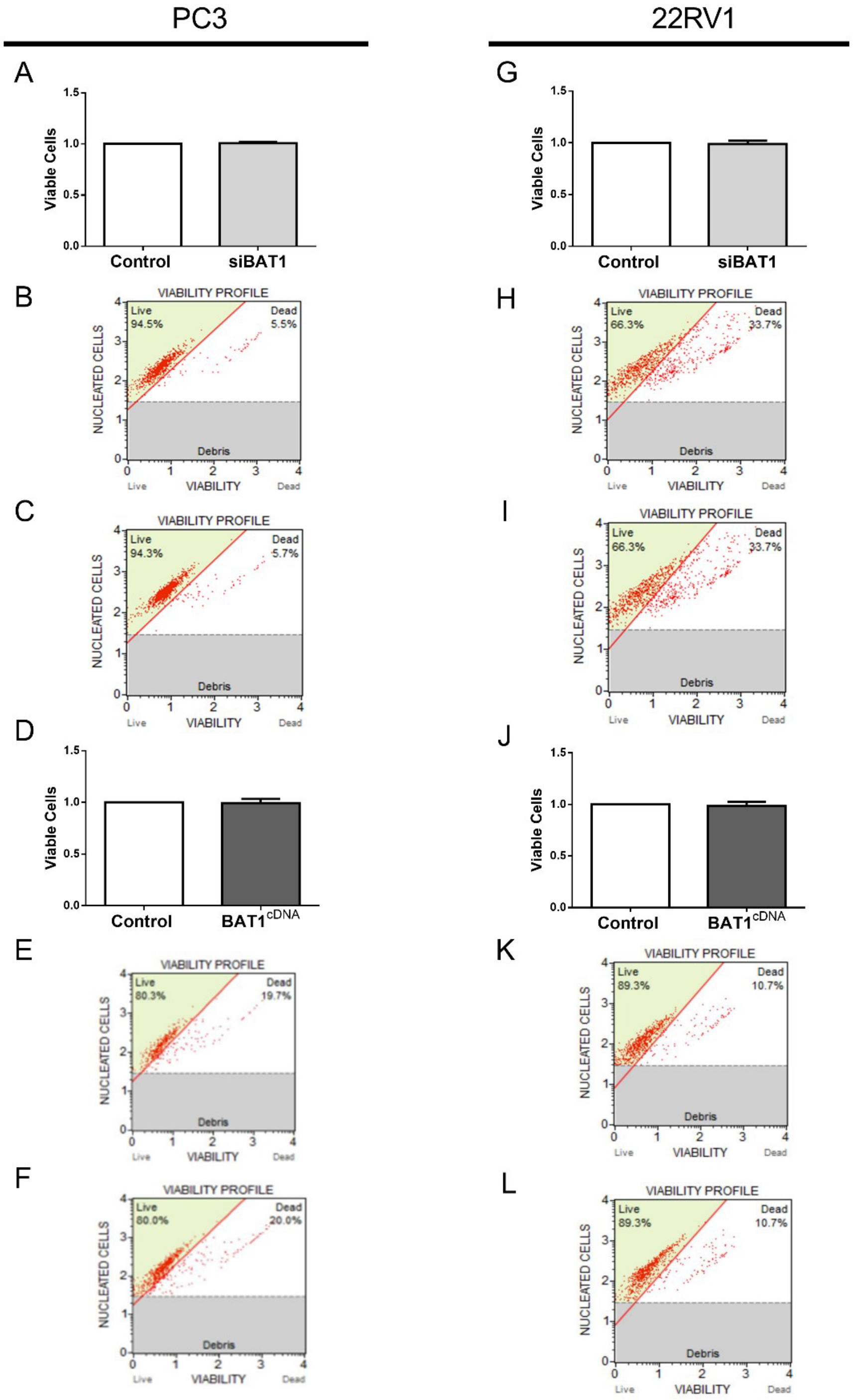

Supplementary Figure 3. BAT1 down-regulation or overexpression showed no change in cell viability in PC3 and 22RV1 cells (A)(D) PC3 cells transfected with siBAT1 or BAT1cDNA showed no significant change in the percentage of viable cells when compared to control. (B)(E) Representative image of control PC3 cells using FACs analysis. (C)(F) Representative image of siBAT1 or BAT1cDNA cells using FACs analysis. (G)(J) 22RV1 cells transfected with siBAT1 or BAT1cDNA showed no change in in the percentage of viable cells when compared to control. (H)(K) Representative image of control 22RV1 cells using FACs analysis. (I)(L) Representative image of siBAT1 or BAT1cDNA 22RV1 cells using FACs analysis.

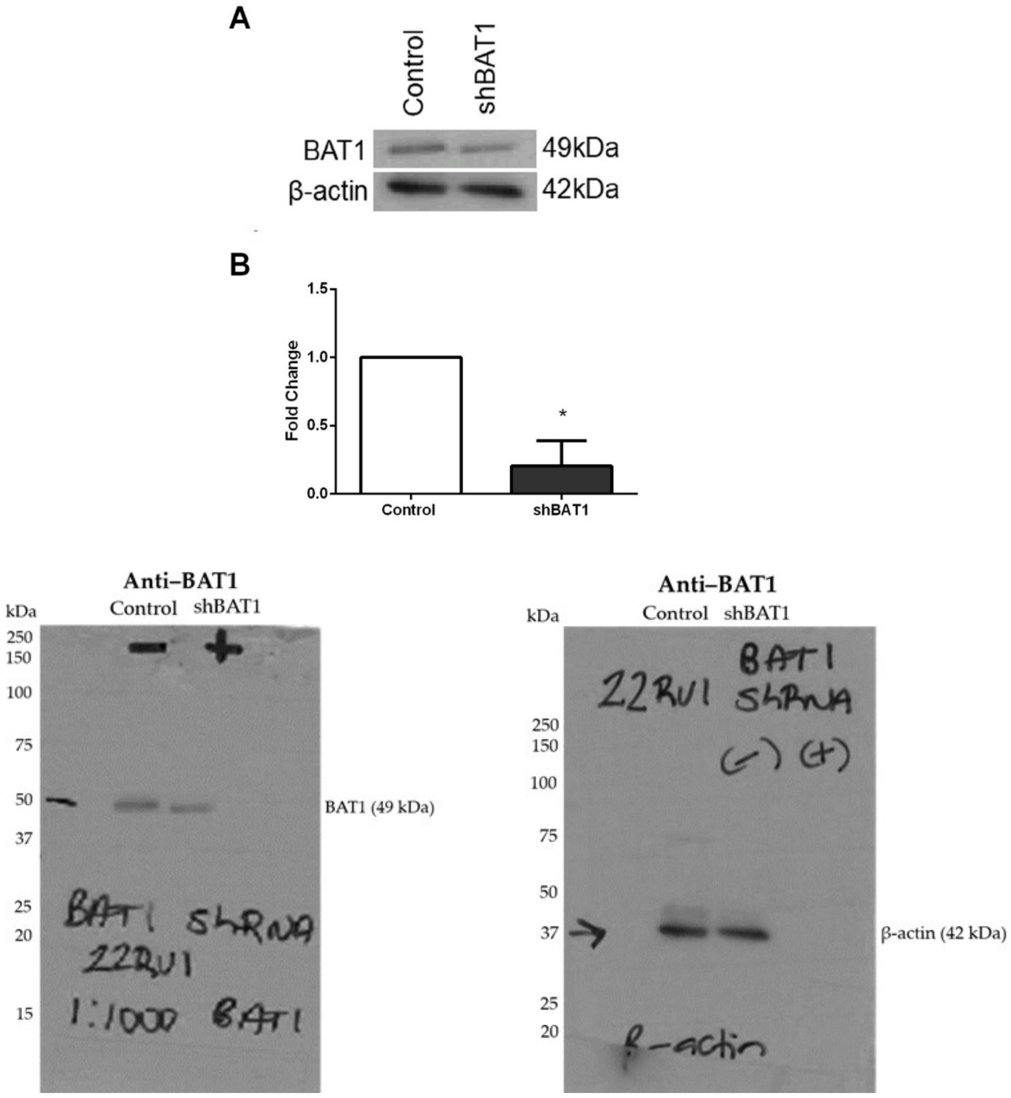

Supplementary Figure 4. BAT1 expression was decreased after shRNA transfection in 22RV1 cells. (A) Representative images of BAT1 protein expression in 22RV1 prostate cancer cell lines using western blot analysis. (B) Quantification of BAT1 protein expression in 22RV1 prostate cancer cell lines using western blot analysis showed a significant decrease in BAT1 protein expression in shBAT1 transfected cells when compared to control (*P<0.05).

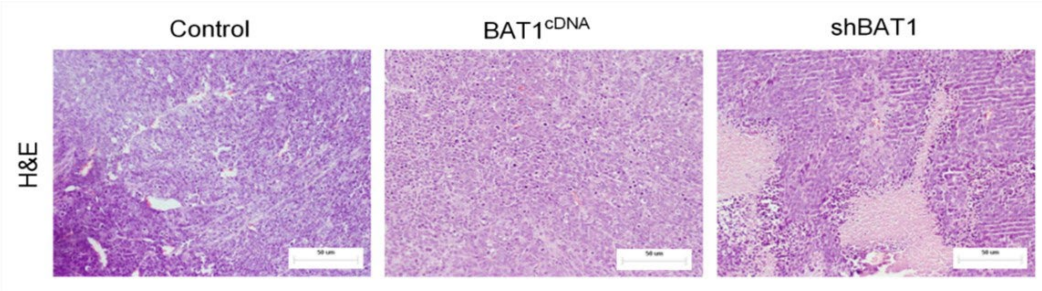

Supplementary Figure 5. BAT1 expressions showed no pathological changes *in vivo*. Representative images of mice prostate tumors using hematoxylin and eosin (H&E) staining.

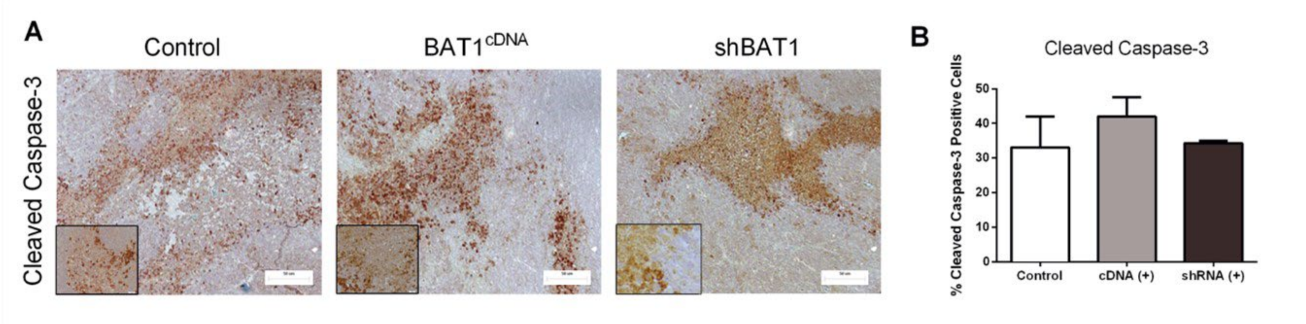

Supplementary Figure 6. BAT1 expressions showed no change in apoptosis *in vivo*. (A) Representative images of mice prostate tumors using immunohistochemistry for the nuclear stained apoptosis marker cleaved caspase 3. (B) Mice prostate tumors with BAT1 expression showed no significant change in cleaved caspase-3 expression when compared to control. Statistical analysis was performed using student's t-test and Mean ± SEM. Scale bars represent: 20X magnification (50μm) and 60X magnification (10μm).

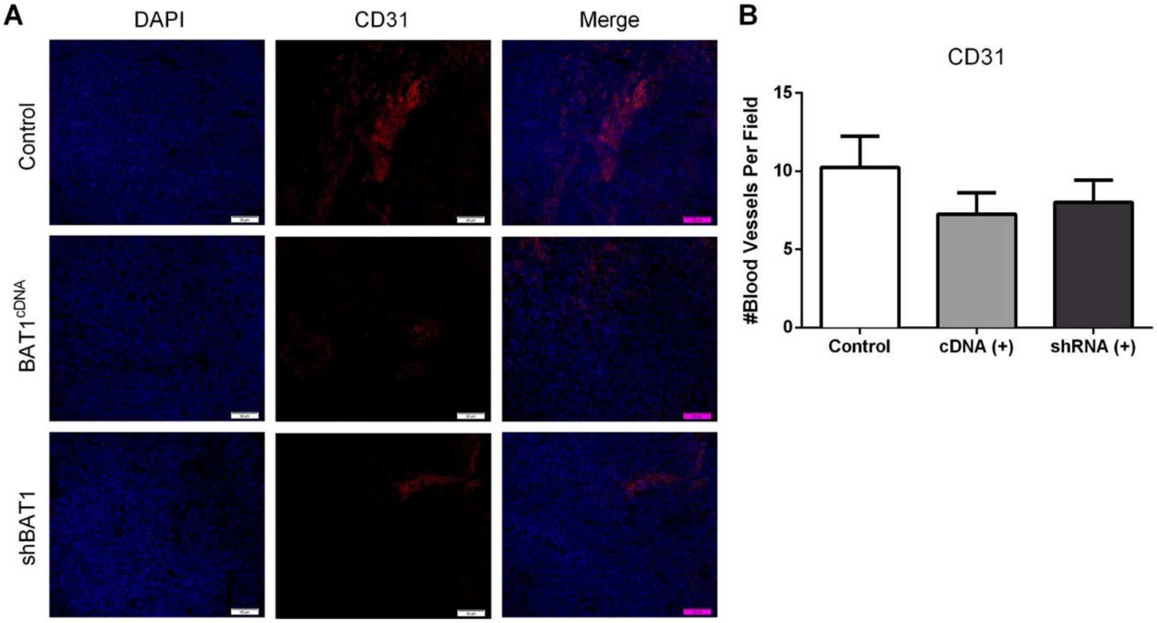

Supplementary Figure 7. BAT1 expressions showed no change in angiogenesis *in vivo*. (A) Representative images of mice prostate tumors using immunofluorescence for the angiogenesis marker CD31. Nuclei are stained in blue (DAPI), and blood vessels (CD31) are stained in red. (B) Mice prostate tumors with BAT1 expression showed no significant change in CD31 expression when compared to control. Statistical analysis was performed using student's t-test and Mean ± SEM. Scale bars represent: 20X magnification (50μm).

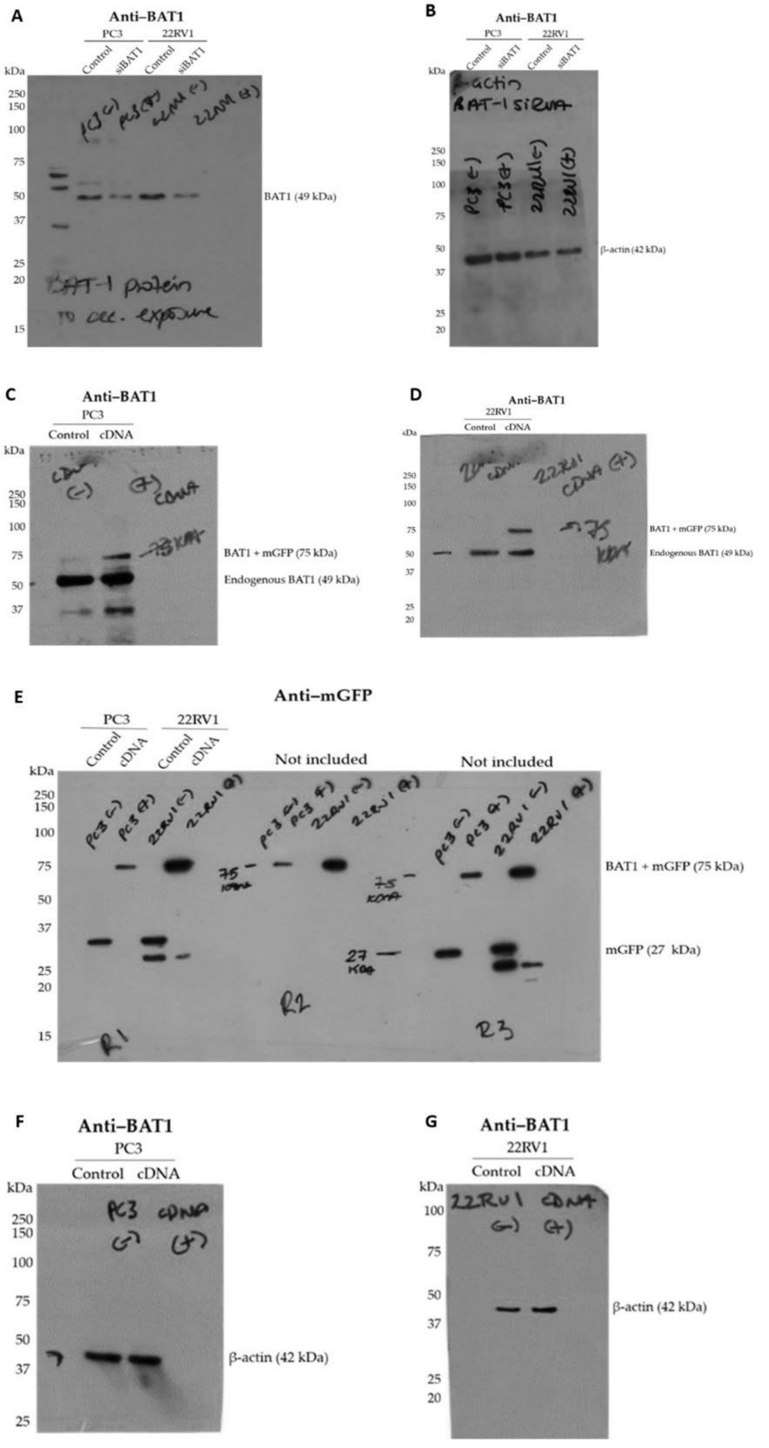

Supplementary Figure 8. Whole western blots images from Figure 1. (A) Representative blot of the BAT1 expression in the 22RV1 and PC3 cells from Figure 1A and 1B. (B) Representative blot of the B-actin expression in the 22RV1 and PC3 cells. (C) Representative blot of BAT1 and endogenous BAT1 expression from Figure 1G. (D) Representative blot of BAT1 and endogenous BAT1 expression from Figure 1H. (E) Representative blot of the expression of the mGFP in 22RV1 and PC3 cells from Figures 1G and 1H. (F) Representation of the expression of B-actin in PC3 cells from Figure 1G. (G) Representation of the expression of B-actin in 22RV1 cells from Figure 1H.

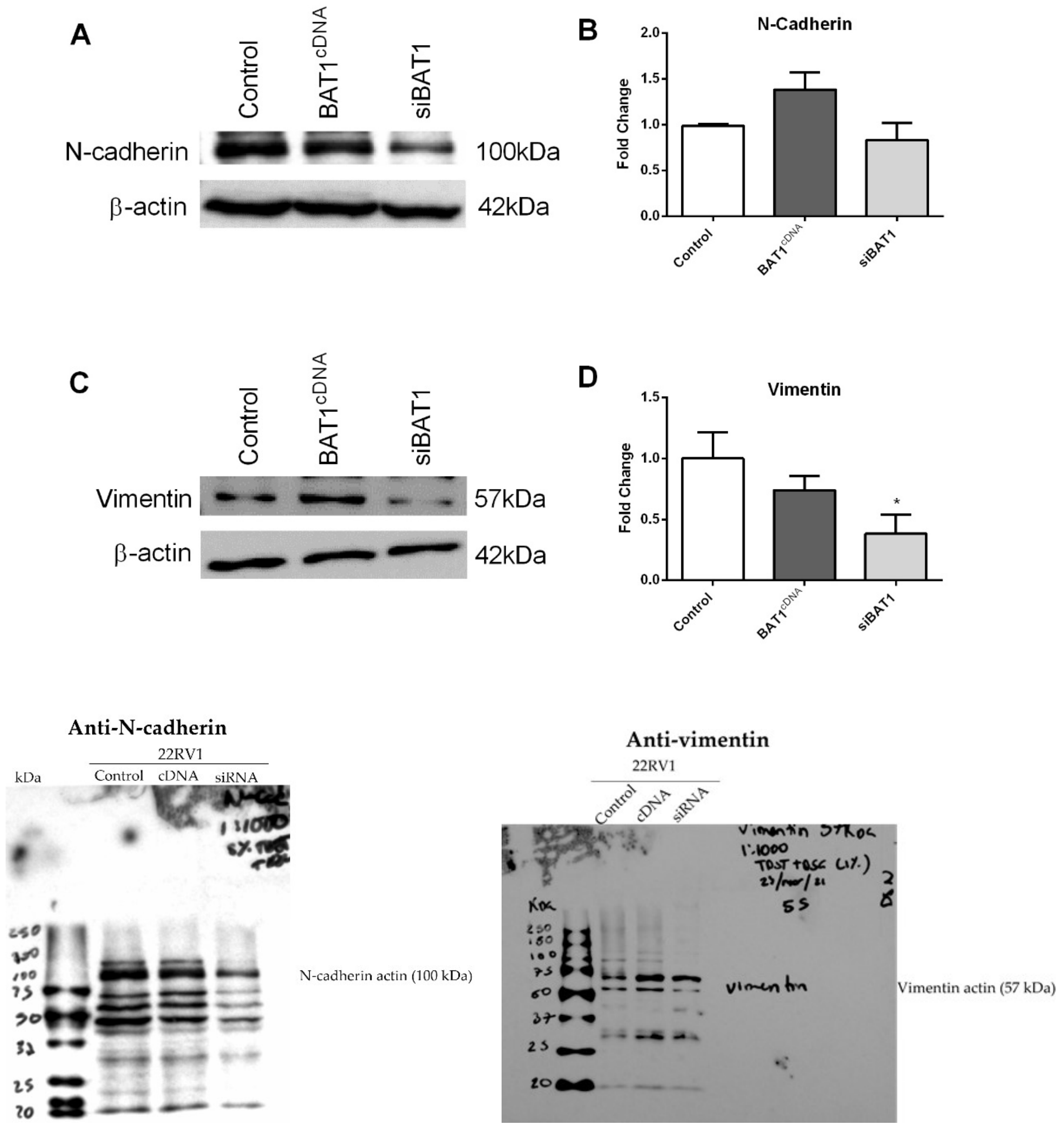

Supplementary Figure 9. Western Blot analysis for N-cadherin and Vimentin. (A) Representative images of N-cadherin protein expression in 22RV1 prostate cancer cells using western blot analysis. (B) Quantification of N-cadherin protein expression using western blot analysis in 22RV1 prostate cancer cell line showed no significant difference in N-cadherin protein expression in BAT1cDNA transfected cells when compared to control. (C) Representative images of Vimentin protein expression in 22RV1 prostate cancer cell line using western blot analysis. (D) Quantification of Vimentin protein expression using western blot analysis in 22RV1 prostate cancer cell line showed a significant decrease in Vimentin protein expression in BAT1cDNA transfected cells when compared to control (*$P<0.01$).

Table S1. Western blot, immunohistochemistry, and immunofluorescence antibody list.

Gene	Company	Catalog Number
BAT1	Epitomics	T3408
IL-6	Novus Biologicals	NBP1-47810
CD31	Abcam	Ab28364
Cleaved Caspase-3	Cell Signaling	9661S
Ki67	Vector	VP-K-451
GFP	Abcam	Ab183734
MMP10	Abcam	Ab199688
TNF-α	Abcam	Ab6671
N-Cadherin	Bioss	Bs-1172-R
Vimentin	Cell Signaling	5741S

Table S2. RT-qPCR primer list.

Gene accession Number	Gene	Forward Primer	Reverse Primer
NM_004640	BAT1	GAAGATGATGAGGTGGAGACAG	TGAGCAGGAAGTCACGAAAG
NM_000594	TNF-α	GATCCCTGACATCTGGAATCTG	GAAACATCTGGAGAGAGGAAGG
NM_0006000	IL-6	GGAGACTTGCCTGGTGAA	CTGGCTTGTTCCTCACTA
NM_002425	MMP10	GGCCCTCTCTTCCATCATATTT	CCTGCTTTACCTCATTTCCT
NM_002427	MMP13	GGAAGAAGAGCTATCAGGAGAAAG	CCAGCCACGCATAGTCATATAG
NM_003255	TIMP2	AGGGCCTGAGAAGGATATAGAG	GGCCTTTCCTGCAATGAGATA
